# Effects of Genetic Variants in the Nicotine Metabolism Pathway on Smoking Cessation

**DOI:** 10.1155/2022/2917881

**Published:** 2022-09-28

**Authors:** Huijie Li, Qiang Wang, Suyun Li, Chongqi Jia

**Affiliations:** ^1^Shandong Provincial Hospital Affiliated to Shandong First Medical University, Jinan, China; ^2^Department of Epidemiology, Shandong University, Jinan, China; ^3^Department of Epidemiology, Weifang Medical University, Weifang, China; ^4^Department of Epidemiology and Health Statistics, Qingdao University, Qingdao, China

## Abstract

**Background:**

We aimed to investigate the associations of various genetic variants in the nicotine metabolism pathway with smoking cessation (SC) in the Chinese Han population.

**Method:**

A case-control study was conducted where 363 successful smoking quitters were referred to as cases, and 345 failed smoking quitters were referred to as controls. A total of 42 genetic variants in 10 genes were selectedand genotyped. The weighted gene score was applied to analyze the whole gene effect. Logistic regression was used to explore associations of each genetic variant and gene score with smoking cessation.

**Results:**

Our study found that the variants *CYP2A6∗4*, rs11726322, rs12233719, and rs3100 were associated with a higher probability of quitting smoking, while rs3760657 was associated with a lower probability of quitting smoking. Moreover, the gene scores of *CYP2D6*, *FMO3*, *UGT2B10*, *UGT1A9*, *UGT2B7,* and *UGT2B15* were shown to exert a positive effect, while the gene score of *CYP2B6* was detected to exert a negative effect on successful smoking cessation.

**Conclusion:**

This study revealed that genetic variants in the nicotine metabolic pathway were associated with smoking cessation in the Chinese Han population.

## 1. Introduction

The usage of tobacco remains the leading cause of preventable death worldwide [1]. It is also associated with an increased risk of various diseases, including respiratory problems, cardiovascular disorders, and cancers [2–4]. In humans, considerable individual variation is observed in the rate of nicotine metabolism, which contributes to differences in various smoking behaviors [5–7]. Compared to the slow metabolizers, a larger amount of cigarette consumption is associated with rapid metabolism of nicotine [8–11], which leads to a higher degree of nicotine dependence [10, 12, 13], increased risk of lung cancer [11, 14, 15], and a lower probability of success in quitting smoking [16–19].

The metabolism of nicotine involves multiple polymorphic catalytic enzymes, whose genetic variabilities were reported to influence nicotine metabolism [20–24]. Considering the importance of nicotine metabolism in SC, we wondered whether genetic variants in the nicotine metabolic pathway contributed to the individual variations in SC. Several studies [16, 25] have reported the relationship between gene variants in genes encoding phase I drug metabolic enzymes, especially *CYP2A6*, and SC. However, to our knowledge, data on the relationship between genetic variants encoding phase II drug metabolic enzymes and smoking behaviors have been lacking so far.

Hence, our study aims to investigate the associations between the genetic variants in the whole nicotine metabolic pathway and SC in the Chinese Han population.

## 2. Methods

### 2.1. Related Definitions

According to the WHO data [26], some of the definitions are as follows: smokers: who have/had smoked 100 or more cigarettes (or the equivalent amount of tobacco) during their lifetime; successful smoking quitters: those who comply with the criterion of smokers but have not smoked at all continuously for two years or more during the survey period [27]; failed smoking quitters: those who comply with the criterion of smokers and had quit smoking but relapsed or did not smoke at all continuously for less than two years during the survey period.

### 2.2. Subjects

This community-based study was conducted among people of 17 villages belonging to three counties (Pingyin, Ju'nan, and Liangshan) of Shandong, China, between April and May 2013. Male participants aged 18 years or more who spontaneously quit smoking anytime were interviewed face to face by well-trained investigators. They were asked to complete a questionnaire designed based on the Global Adult Tobacco Survey (GATS) Core Questionnaire with optional questions. All subjects recruited were of Han Chinese ethnicity. A total of 708 blood samples, including 363 for successful smoking quitters and 345 for failed smoking quitters, were collected successfully and genotyped. Baseline characteristics of all the subjects are provided in Table 1.

In this study, a community-based case-control analysis was performed, where successful smoking quitters were referred to as cases, and failed smoking quitters were referred to as controls. This study was approved by the Ethics Review Committee of Shandong University, where all subjects provided informed consent.

### 2.3. Selection of the Genetic Variants

The genetic variants in this study were selected based on the following criteria:

(1) Identification of TagSNPs by Haploview software 4.2 based on the Chinese Han Beijing (CHB) population data of HapMap (HapMap Data Rel 27 Phase II + III, Feb 09, on NCBI B36 assembly, dbSNP b126). TagSNPs were selected if pairwise *r*^2^ > 0.8 and minor allele frequency (MAF) > 0.05. (2) Functional relevance and commonality [MAF> 0.05, based on the CHB population data of the dbSNP database and 1000 Genomes (https://browser.1000.genomes.org/index.html)]. (3) Genetic variants showing a significant relationship with smoking behaviors in previous studies. Overall, a total of 41 SNPs and one gene deletion allele among 10 genes were selected.  Table 2 presents detailed information on these 42 genetic variants.

### 2.4. SNPs Genotyping

Blood samples were used to extract genomic DNA by a DNA isolation kit (TIANGEN, China). Next, the SNP genotypes were determined using the Matrix-Assisted Laser Desorption/Ionization Time of Flight Mass Spectrometry (MALDI-TOF–MS) of the MassARRAY system (Sequenom Inc., San Diego, CA, USA). The Assay Design 3.1 software (Sequenom Inc., San Diego, CA, USA) was used to design PCR primers. Genotyping was performed by the Bio Miao Biological Technology (Beijing) Co., Ltd., without any knowledge about the case or control status.

### 2.5. CYP2A6 Genotyping

The presence of *CYP2A6∗∗4* (whole gene deletion) was detected by a two-step allelic-specific PCR assay [14, 28]. The first PCR reaction (PCR I) was performed using the primers 2Aex7F and 2A6R1 (Table 3). All primers were all synthesized by Sangon Biotech Co., Ltd. (Shanghai, China). The total reaction mixture (50 *μ*l) included 4 *μ*l of genomic DNA, 1.5 *μ*l of each primer (10 *μ*M), 25 *μ*l of 2 × Taq PCR Master Mix (BBI), and 18 *μ*l of ddH_2_O. The PCR program was as follows: initial denaturation step at 94°C for 4 min, followed by 35 cycles of denaturation at 94°C for 30 s, annealing at 56°C for 30 s, extension at 72°C for 2.5 min, and then a final extension at 72°C for 10 min.

Next, the allele-specific PCR reaction was performed (PCR II), which involved a PCR mixture containing 2 *μ*L of PCR I product, 2 *μ*L of primer 2A6ex8F (10 *μ*M) or primer 2A7ex8F (10 *μ*M), 2 *μ*L of primer 2A6R2, 25 *μ*L of 2 × Taq PCR Master Mix (BBI), and 19 *μ*L of ddH_2_O, making up a total volume of 50 *μ*L. The amplification was performed as per the following program: initial denaturation at 94°C for 4 min, followed by 30 cycles of denaturation at 94°C for 30 s, annealing at 52°C for 45 s, extension at 72°C for 1.5 min, and then a final extension at 72°C for 10 min. The amplified PCR products were analyzed on a 1.5% agarose gel (BBI) and stained with ethidium bromide.

As per the definition by Tamaki et al. [14], the CYP2A-specific 1,181-bp product amplified using the 2A6ex8F/2A6R2 primer pair alone indicated the presence of wildtype *CYP2A6* (*CYP2A6 non* *∗* *4/non* *∗* *4*). Similarly, the product amplified using the primer pair 2A7ex8F/2A6R2 alone indicated a *CYP2A6 deletion* (*CYP2A6*∗*4*/∗*4*∗). However, if one individual sample showed product amplification in both reactions, it indicated heterozygosity (*CYP2A6 non* *∗* *4/∗4*).

### 2.6. Construction of the Weighted Gene Score

To increase the power of the test and explore the effect of the whole genes on SC, the weighted gene score of each gene was calculated as the sum of each genotype multiplied by its weight, which was then divided by the sum of the weights [29, 30]. The risk score was calculated as per the following equation:

(1) $$\begin{array}{c} \text{weighted gene score}=\frac{\left(w_{1}\times {\text{SNP}}_{1}+w_{2}\times {\text{SNP}}_{2}+\cdots +w_{k}\times {\text{SNP}}_{k}\right)}{\left(w_{1}+w_{2}+\cdots +w_{k}\right)}, \end{array}$$

where SNP_*i*_ has a value of 0, 1, or 2 according to the number of minor alleles for SNP; *W*_k_ is the weight of SNP calculated using logistic regression, where smoking cessation is the dependent variable, and each variant is the independent variable; *K* is the number of SNPs in each gene [29, 31]. Since we analyzed only one genetic variant in the *CYP2A6* gene (*CYP2A6∗4*), we did not calculate the gene score of *CYP2A6*.

### 2.7. Statistical Analysis

To describe the demographic characteristics of participants, the frequency and percentage of categorical variables were calculated along with the mean and standard deviation of metric variables. Additionally, Pearson's *χ*^2^ test was used to compare the differences between the categorical variables of the case and control groups, while the Hardy-Weinberg Equilibrium (HWE) was used to compare differences among the control groups. If the variance between the groups was homogeneous, a one-way analysis of variance was used to test the differences in means of metric variables between the groups. Otherwise, the Kruskal-Wallis equality-of-populations rank test was used.

The associations between the genetic variants and SC were evaluated using the odds ratios (ORs) and 95% confidence intervals (CIs), which were first calculated using univariate logistic regression, and then by multiple logistic regression analysis with adjustments being done for age, occupation, education level, marital status, age of smoking onset and pack-year. The association studies were analyzed among four genetic models, including codominant, additive, dominant, and recessive models. Furthermore, the Akaike information criterion (AIC) was utilized to determine the best genetic model for each SNP.

The complete gene effect on SC was analyzed using the logistic regression, where odds ratios (ORs) and multivariate-adjusted ORs (adjusted for age, occupation, education level, marital status, age of smoking onset, and pack-year) were calculated for each gene score and smoking cessation.

To estimate the value of power, we used a range of minor allele frequencies (MAF) in genetic variants, including 0.1, 0.2, 0.3, 0.4, and 0.5. We assumed an odds ratio (OR) of 1.5, the population risk of 11.7% [32], and the type I error rate (*α*) of 0.05. Our results showed that a total sample size of 708 subjects, including 363 cases and 345 controls, provided a power of more than 80% for 0.2, 0.3, 0.4, and 0.5 of MAF and more than 60% power for 0.1 of MAF under the additive inheritance model.

While power calculation was performed using the program QUANTO 1.2.4, the other statistical analyses were carried out using the STATA/SE version 15.1 (Stata Corporation, College Station, TX, USA). All reported probabilities (*P* value) were two-sided, and a *P* value less than 0.05 was considered statistically significant.

## 3. Results

### 3.1. Participant Characteristics

The demographic characteristics of both the groups, including 363 successful smoking quitters (cases) and 345 failed smoking quitters (controls), are summarized in Table 1. Compared to the failed smoking quitters, the successful smoking quitters showed significantly higher age and shorter smoking duration (*P* < 0.001). Moreover, the distribution of the marital status between the case and control groups showed significant differences (*P* < 0.05).

### 3.2. Associations of Genetic Variants in the Nicotine Metabolic Pathway with Smoking Cessation

**Figure 1:**
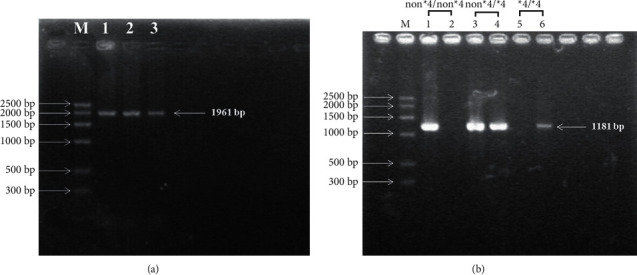
(a) First-step PCR results (1,961-bp) (b) second-step PCR results (1,181-bp). Lanes 1 and 2 are wildtype *CYP2A6* (non *∗* 4/non *∗* 4); lanes 3 and 4 are heterozygous deletions (non *∗* 4/*∗*4); lanes 5 and 6 are the *CYP2A6* deletion genotype (*∗*4/*∗*4).

Table 2 lists the minor allele frequency (MAF) for each genetic variant in all the subjects, along with the *P* value of the control subjects from the HWE test.  Figure 1 presents the genotyping assay results obtained from the two-step allelic-specific PCR assay.

The frequencies of the genotype and the ORs (95% CI) for the codominant, additive, dominant, and recessive models are presented in Supplementary Table 1. The univariate analyses showed that *CYP2A6∗4* was correlated with an increased possibility of SC in the codominant [∗4/*∗*4 vs non *∗* 4/non *∗* 4, 2.016 (1.270–3.201)], additive [non *∗* 4/non *∗* 4 vs non *∗*4/*∗*4 vs *∗*4/*∗*4, 1.353 (1.095–1.672)], dominant [non *∗*4/*∗*4 + *∗*4/*∗*4 vs non *∗* 4/non *∗* 4, 1.442 (1.041–1.998) ], and recessive models [*∗*4/*∗*4 vs non *∗* 4/non *∗* 4 + non *∗* 4/*∗*4, 1.976 (1.252–3.117)]. Besides, rs11726322 of the *UGT2B10* gene, rs12233719 of the *UGT2B7* gene, and rs3100 of the *UGT2B15* gene were also found to exert a protective effect on SC among different genetic models. However, a negative association was detected between rs3760657 of *CYP2B6* gene and SC in the codominant [AG vs AA, 0.669 (0.488–0.918)], additive [AA vs AG vs GG, 0.773 (0.599–0.997)], and dominant [AG + GG vs AA, 0.691 (0.599–0.936)] genetic models.

The multiple logistic regression analysis, adjusted for the potential confounders, showed significant associations of *CYP2A6∗4*, rs11726322, rs12233719, and rs3100 with the increased possibility of SC, as well as rs3760657 with the reduced possibility of SC. Based on the Akaike information criterion (AIC), the corresponding optimal recessive model was *CYP2A6∗4*, rs11726322, and rs12233719, while the optimal dominant model was rs3760657 and the additive model was rs3100. However, no significant relationship was found between other genetic variants and SC among the four genetic models.

### 3.3. Association between the Gene Scores and Smoking Cessation

The results of the association between the gene scores and SC are presented in Table 4. Positive significant associations were observed between the gene scores of *CYP2D6*, *FMO3*, *UGT2B10*, *UGT1A9*, *UGT2B7,* and *UGT2B15* and SC. However, a negative association was found between *CYP2B6* gene score and SC. The results remained significant even after adjusting for age, occupation, education level, marital status, and age of onset of smoking. Moreover, the total gene score for all the selected genetic variants in the nicotine metabolic pathway showed a positive association with SC both before (OR = 3.311, 95% CI: 2.342–4.679) and after adjusting the above-mentioned potential confounders (OR = 3.411, 95% CI: 2.382–4.884).

## 4. Discussion

Smoking behavior is a complex trait with a multigenic etiology, which is influenced by both environmental and genetic factors. A genetic influence with heritability has been identified in smoking cessation (SC), which is estimated at 50–58% [33–36]. In this study, we investigated the associations between various genetic variants in the nicotine metabolic pathway and SC in the Chinese Han population. Our results showed that *CYP2A6∗4*, rs11726322, rs12233719, and rs3100 were associated with a higher probability while rs3760657 was associated with a lower probability of quitting smoking. Moreover, the gene scores of *CYP2D6*, *FMO3*, *UGT2B10*, *UGT1A9*, *UGT2B7,* and *UGT2B15* were shown to exert a positive effect on successful SC, while the gene score of *CYP2B6* was detected to exert a negative effect.

Nicotine is primarily metabolized by the following three pathways: cytochrome P450 (CYPs)-catalyzed C-oxidation, UDP-glucuronosyltransferases (UGTs)-catalyzed glucuronidation, and flavin-containing monooxygenase 3 (FMO3)-catalyzed *N*-oxidation [37]. In smokers, 70–80% of nicotine is converted to cotinine before metabolization to other metabolites [38]. The C-oxidation of nicotine to cotinine occurs via a two-step mechanism, where nicotine is first oxidized to the nicotine-Δ-1′ (5′)-iminium ion mediated by CYPs, which is followed by its conversion to cotinine by cytosolic aldehyde oxidase 1 (AOX1) [39]. Subsequently, cotinine is further oxidized by CYPs to *trans*-3′-hydroxycotinine (3HC), which accounts for 27–40% of the nicotine dose recovered in urine. This is the main nicotine metabolite detected in the urine of smokers [39, 40]. CYP2A6 is the major CYP enzyme involved in the C-oxidation of nicotine. However, in some individuals, other CYPs, including CYP2B6 and CYP2D6, also contribute minorly [41–43]. The gene encoding the CYP2A6 enzyme is highly polymorphic and results in extensive interindividual variations in the CYP2A6 enzyme activity, affecting the rate of metabolism of nicotine [25, 44]. Nakajima et al. [45] first reported that the poor metabolism of nicotine to cotinine was attributed to the whole deletion of the *CYP2A6* gene (*CYP2A6∗4*) in humans. Compared to other ethnic populations, the *CYP2A6∗4* was reported to have a high allelic frequency in Asians (11–24%) [21, 28, 46, 47]. In this study, *CYP2A6∗4* was correlated with a higher probability of quitting smoking. However, the deviation from HWE in the control group for *CYP2A6∗4* was observed in our study; our result may be biased and needs to be further confirmed.

For in vitro C-oxidation of nicotine, CYP2B6 is responsible for an approximately 10% catalytic efficiency of the CYP2A6 enzyme [48]. While CYP2A6 is primarily expressed in the liver, CYP2B6 is expressed at higher levels in the brain. Higher brain activity for CYP2B6 may cause altered sensitivity to centrally acting drugs, which may contribute to influencing the localized metabolism of nicotine in the brains of human smokers [49].

Approximately 4–7% of absorbed nicotine is excreted in the urine as nicotine *N*′-oxide [50, 51], which is converted by flavin-containing monooxygenase 3 (FMO3) [46, 52]. A common decrease in the function of *FMO3* alleles exerts modest effects on *N*-oxidation activity caused by a slight modulation of protein levels and/or function and is more likely to contribute to general population variation in FMO3 [53]. In 2014, Chenoweth et al. [54] showed that *FMO3* E158K (rs2266782) modestly influenced the systemic nicotine metabolism within the *CYP2A6* subgroups. However, in this study, we did not observe any significant relationship between *FMO3* alleles and SC.

Nicotine, cotinine, and *trans*-3′-hydroxycotinine undergo further phase II detoxification reactions catalyzed by the UDP-glucuronosyltransferase (UGTs) family of enzymes via the conjugation with glucuronic acid [55]. In smokers, 3–5% of absorbed nicotine is excreted in the urine as nicotine *N*-glucuronide, 16–17% as cotinine *N*-glucuronide, as well as an additional 8–9% as *trans*-3′-hydroxycotinine-*O*-glucuronide [50, 51]. The UDP-glucuronosyltransferases (UGTs) include a large family of conjugation enzymes, detoxifying a wide variety of both endogenous and exogenous substrates [56]. Kuehl and Murphy [57] reported that UGT1A4 and UGT1A9, the UGTs isoforms, were responsible for nicotine and cotinine *N*-glucuronidation. Although glucuronidation of t*rans*-3′-hydroxycotinine to *O*-glucuronide occurs mainly via UGT2B7 and UGT2B10 [58], it is partly glucuronidated by UGT1A9 and UGT2B15 [59]. A change in the gene encoding enzymes (i.e., point mutation, deletion, and gene conversion) responsible for chemical metabolism may lead to overproduction, underproduction, malfunction, or absence of the protein, finally resulting in alterations in the functioning of the enzyme [39]. Many *UGT* gene variants were reported to influence the glucuronidation of tobacco-related compounds, including *UGT1A4* [60, 61], *UGT2B7* [62], and *UGT2B10* [62, 63].

Several studies have reported a significant impact of nicotine metabolism on the probability of success in quitting smoking [16–19]. Evidence suggests that smokers adapt their smoking behavior to maintain desired levels of nicotine in the body. The level of nicotine in the body is determined according to the intake of nicotine and the rate of metabolism by the liver. Based on the above-mentioned associations between genetic polymorphisms and enzymatic activity, we infer that genetic variants in the nicotine metabolic pathway may contribute to the individual variability in SC.

In our study, we not only analyzed single variants but also used gene scores to analyze the whole effect of the genes in the nicotine metabolic pathway on SC. A gene score is important to model multifactorial polygenic traits, particularly when the gene score consists of many common variants with small effects [29]. The gene score may explain a considerable proportion of variations in the risk factor, even when none of the variants can explain it individually, thus, making it a very popular method for genetic association studies [29].

One of the strengths of our study is the exploration of the relationship between the gene variants encoding phase II drug metabolic enzymes, including *UGT1A9*, *UGT2B10*, and *UGT2B7,* and smoking behavior. To our knowledge, data on such a relationship is rarely available so far. Most researchers have focused on genes encoding phase I drug metabolic enzymes, especially *CYP2A6*. Our study reveals that rs11726322 of *UGT2B10*, rs12233719 of *UGT2B7,* and rs3100 of *UGT2B15* may influence the susceptibility of SC, which has not been reported in previous publications. Additionally, we employed the method of gene score for the association analysis, which increased the power and simplicity of the test.

However, we acknowledge some limitations in our study. Firstly, since smoking behaviors have a complex etiology generated by many factors, some other uninvestigated variables may alter the results. Secondly, this was a community-based case-control study and may have some biases, including selection and recall bias, which may distort the results.

## 5. Conclusions

This study revealed that five single variants (*CYP2A6∗4*, rs11726322, rs12233719, rs3100, and rs3760657) and gene scores of *CYP2B6*, *CYP2D6*, *FMO3*, *UGT2B10*, *UGT1A9*, *UGT2B7*, and UGT2B15 might influence the susceptibility of SC in the Chinese Han population. Further examinations using larger sample size and other ethnic groups may be required to confirm our findings.

## Figures and Tables

**Table 1:** The characteristics of the subjects.

Variables	Successful quitters (*n* = 363)	Failed quitters (*n* = 345)	*P*
Age (years)	61.931 ± 10.806	57.499 ± 12.090	*P* < 0.001
Age group (young/middle/old)	22 (6.06)/188 (51.79)/153 (42.15)	51 (14.78)/194 (56.23)/100 (28.99)	*P* < 0.001
Occupation (farmer/others)	289 (76.61)/74 (20.39)	276 (80.00)/69 (20.00)	0.898
Education (low/middle/high)	111 (30.58)/204 (56.20)/48 (13.22)	85 (24.64)/211 (61.16)/49 (14.20)	0.210
Marital status (married/unmarried/others)	319 (87.88)/7 (1.93)/37 (10.19)	322 (83.33)/3 (0.87)/20 (5.80)	0.044
Age of smoking onset (year)	21.375 ± 5.569	22.296 ± 7.049	0.054
CPD	21.333 ± 15.864	19.316 ± 11.545	0.055
Pack-year	31.458 ± 27.658	32.215 ± 23.636	0.696
Smoking duration (years)	29.026 ± 13.088	33.693 ± 13.377	*P* < 0.001

Values are expressed as mean ± SD or frequency (%). Age group: young = age <45, middle = 45≤ age <65, old = age ≥65. Education: low = below the primary school, middle = primary and middle school, high = high school or more. Pack-year = CPD/20 × number of years smoked. *CPD*: cigarettes per day.

**Table 2:** Genetic variants investigated in this study.

Gene	SNP ID	Chr	Chr position (GRCh38.p2)	Alleles (major/minor)	MAF (case/control)	HWE *P*^a^
*CYP2B6*	rs3760657	19	40989528	A/G	0.197/0.241	0.242
	rs4802101	19	40990556	C/T	0.366/.0348	0.951
	rs707265	19	41018182	G/A	0.391/0.378	0.821

*CYP2D6*	rs1135840	22	42126611	G/C	0.326/0.306	*P* < 0.001
	rs16947	22	42127941	G/A	0.176/0.187	0.004
	rs1081003	22	42129754	G/A	0.484/0.484	0.370
	rs1065852	22	42130692	G/A	0.467/0.499	0.258

*AOX1*	rs2072034	2	200585011	C/G	0.393/0.435	0.114
	rs10931910	2	200659013	G/A	0.136/0.130	0.375
	rs3731722	2	200669666	A/G	0.088/0.078	0.508

*FMO3*	rs1736555	1	171089111	G/A	0.375/0.413	0.358
	rs10911192	1	171102742	A/C	0.452/0.429	0.914
	rs2266782	1	171107825	G/A	0.225/0.193	0.271
	rs1736557	1	171110939	G/A	0.209/0.212	0.616
	rs2075992	1	171111344	T/C	0.349/0.370	0.370
	rs909529	1	171113756	C/T	0.237/0.216	0.354
	rs909530	1	171114034	C/T	0.401/0.374	0.957
	rs2266780	1	171114102	A/G	0.192/0.181	0.331
	rs909531	1	171114397	T/C	0.196/0.186	0.141

*UGT1A4*	rs3806594	2	233717244	T/C	0.174/0.191	0.107
	rs3732217	2	233719624	G/A	0.167/0.191	0.107

*UGT2B10*	rs2942857	4	68822269	A/C	0.090/0.097	0.042
	rs11726322	4	68825803	G/C	0.183/0.151	0.233
	rs4694358	4	68830930	T/C	0.135/0.120	0.011

*UGT1A9*	rs17864684	2	233670717	G/A	0.132/0.146	0.144
	rs7349250	2	233673274	A/G	0.231/0.217	0.173
	rs2602379	2	233674416	G/A	0.464/0.438	0.450
	rs1604144	2	233697189	C/T	0.238/0.254	0.533
	rs12988520	2	233698748	A/C	0.278/0.268	0.154
	rs2885295	2	233711220	T/A	0.175/0.187	0.032
	rs871514	2	233719883	T/C	0.200/0.167	*P* < 0.001
	rs10178992	2	233749231	T/A	0.150/0.125	0.502
	rs10929303	2	233772770	C/T	0.145/0.132	0.638

*UGT2B7*	rs12233719	4	69096731	G/T	0.175/0.138	0.012
	rs7439366	4	69098620	C/T	0.362/0.384	0.800
	rs12512526	4	69100700	C/T	0.360/0.386	0.606
	rs4292394	4	69107231	G/C	0.361/0.389	0.490

*UGT2B15*	rs3100	4	68646936	G/A	0.203/0.144	0.694
	rs4148269	4	68647129	T/G	0.194/0.165	0.537
	rs9994887	4	68671757	A/G	0.493/0.483	0.612
	rs13112099	4	68672015	T/G	0.320/0.332	*P* < 0.001

*CYP2A6*	*CYP2A6 deleted ( * ^*∗*^ * 4)*	19	Whole gene	Non *∗* 4/*∗*4	0.249/0.174	*P* < 0.001

*MAF*: minor allele frequency; *HWE*: Hardy-Weinberg Equilibrium. ^a^The HWE *P* value shows the Hardy-Weinberg Equilibrium test in control subjects.

**Table 3:** Primers used for amplification of *CYP2A6∗4* (gene deletion).

Reaction	Primer name	Primer sequence	Product size (bp)
PCR I	2Aex7F	5′-CCAAGATGCCCTACATG-3′	1,967
	2A6R1	5′-CTTATGTTTTGTGAGACATCAGAGACAA-3′	

PCR II	2A6ex8F	5′-CACTTCCTGACTGAG-3′	1,180
	2A7ex8F	5′-CATTTCCTGGATGAC-3′	
	2A6R2	5′-AAAATGGGCATGAACGCCC-3′	

*F* forward primer, *R* reverse primer.

**Table 4:** Association between the gene scores and smoking cessation.

Gene	Successful quitters (*n* = 363)	Failed quitters (*n* = 345)	OR (95% CI)	*P*	OR (95% CI)^a^	*P* ^*a*^
*CYP2B6*	0.394 ± 0.598	0.485 ± 0.597	0.776 (0.605–0.994)	0.045	0.729 (0.563–0.944)	0.016
*CYP2D6*	1.344 ± 0.326	1.293 ± 0.327	1.618 (1.021–2.562)	0.040	1.678 (1.038–2.713)	0.035
*AOX1*	1.278 ± 1.246	1.445 ± 1.286	0.901 (0.802–1.013)	0.081	0.903 (0.801–1.019)	0.098
*FMO3*	0.343 ± 0.668	0.206 ± 0.598	1.410 (1.109–1.792)	0.005	1.405 (1.096–1.799)	0.007
*UGT1A4*	0.256 ± 0.947	0.383 ± 0.843	0.848 (0.710–1.013)	0.069	0.873 (0.728–1.047)	0.144
*UGT2B10*	0.353 ± 0.436	0.290 ± 0.387	1.447 (1.010–2.073)	0.044	1.489 (1.026–2.159)	0.036
*UGT1A9*	16.767 ± 22.785	11.698 ± 20.891	1.011 (1.004–1.018)	0.002	1.011 (1.004–1.019)	0.002
*UGT2B7*	0.228 ± 0.784	0.107 ± 0.701	1.245 (1.020–1.520)	0.032	1.237 (1.006–1.521)	0.044
*UGT2B15*	0.546 ± 0.720	0.378 ± 0.638	1.444 (1.155–1.807)	0.001	1.466 (1.162–1.851)	0.001
Total	0.703 ± 0.479	0.451 ± 0.440	3.311 (2.342–4.679)	*P* < 0.001	3.411 (2.382–4.884)	*P* < 0.001

Values are expressed as mean ± SD. ^a^ indicates adjustment for age, occupation, education level, marital status, age of smoking onset, and pack-year.

## Data Availability

All data generated or analyzed during this study are included within the article and its supplementary information files.

## Supplementary Materials

Supplementary Materials Supplementary Table 1: associations of genetic variants in nicotine metabolism pathway with smoking cessation under different genetic models.
