# Supplementary material for: Effects of Genetic Variants in the Nicotine Metabolism Pathway on Smoking Cessation
Source: Genet Res (Camb). 2022 Sep 28;2022:2917881. doi: 10.1155/2022/2917881 (PMC9534651; doi:10.1155/2022/2917881)
Supplement: Supplementary Materials — Supplementary Table 1: associations of genetic variants in nicotine metabolism pathway with smoking cessation under different genetic models. [file 2917881.f1.docx]

| **Supplementary Table 1** Associations of genetic variants in nicotine metabolism pathway with smoking cessation under different genetic models | | | | | | | | | | |
| --- | --- | --- | --- | --- | --- | --- | --- | --- | --- | --- |
| Gene | SNP ID | Model |  | Successful quitters | Failed quitters | OR (95%CI) | *P* | OR (95%CI)*^a^* | *P^a^* | AIC*^a^* |
| *CYP2A6* | *CYP2A6*4* | Codominant | non *4/ non *4 | 243 (66.94) | 257 (74.49) | 1.0 (Ref.) | – | 1.0 (Ref.) | – | 954.969 |
|  |  |  | non *4/*4 | 59 (16.25) | 56 (16.23) | 1.114 (0.743–1.672) | 0.601 | 1.091 (0.718–1.659) | 0.684 |  |
|  |  |  | *4/*4 | 61 (16.80) | 32 (9.28) | 2.016 (1.270–3.201) | 0.003 | 1.955 (1.212–3.154) | 0.006 |  |
|  |  | Additive | non *4/ non *4 vs non *4/*4 vs *4/*4 | | | 1.353 (1.095–1.672) | 0.005 | 1.330 (1.069–1.656) | 0.011 | 954.150 |
|  |  | Dominant | non *4/ non *4 | 243 (66.94) | 257 (74.49) | 1.0 (Ref.) | – | 1.0 (Ref.) | – | 956.871 |
|  |  |  | non *4/*4 + *4/*4 120 (33.06) | | 88 (25.51) | 1.442 (1.041–1.998) | 0.028 | 1.405 (1.003–1.967) | 0.048 |  |
|  |  | Recessive^*^ | non *4/ non *4 + non*4/*4 302 (83.20) | | 313 (90.72) | 1.0 (Ref.) | – | 1.0 (Ref.) | – | 953.135 |
|  |  |  | *4/*4 | 61 (16.80) | 32 (9.28) | 1.976 (1.252–3.117) | 0.003 | 1.924 (1.200–3.085) | 0.007 |  |
| *CYP2B6* | rs3760657 | Codominant | AA | 237 (65.29) | 195 (56.52) | 1.0 (Ref.) | – | 1.0 (Ref.) | – | 955.021 |
|  |  |  | AG | 109 (30.03) | 134 (38.84) | 0.669 (0.488–0.918) | 0.013 | 0.630 (0.454–0.874) | 0.006 |  |
|  |  |  | GG | 17 (4.68) | 16 (4.64) | 0.874 (0.430–1.755) | 0.710 | 0.775 (0.370–1.623) | 0.500 |  |
|  |  | Additive | AA vs AG vs GG | |  | 0.773 (0.599–0.997) | 0.047 | 0.726 (0.557–0.946) | 0.018 | 955.144 |
|  |  | Dominant^*^ | AA | 237 (62.59) | 195 (56.52) | 1.0 (Ref.) | – | 1.0 (Ref.) | – | 953.312 |
|  |  |  | AG + GG | 126 (34.71) | 150 (43.48) | 0.691 (0.599–0.936) | 0.017 | 0.645 (0.471–0.884) | 0.006 |  |
|  |  | Recessive | AA + AG | 346 (95.32) | 329 (95.36) | 1.0 (Ref.) | – | 1.0 (Ref.) | – | 960.754 |
|  |  |  | GG | 17 (4.68) | 16 (4.64) | 1.010 (0.502–2.033) | 0.977 | 0.921 (0.445–1.905) | 0.824 |  |
|  | rs4802101 | Codominant | CC | 140 (38.57) | 147 (42.61) | 1.0 (Ref.) | – | 1.0 (Ref.) | – | 960.144 |
|  |  |  | TC | 180 (49.59) | 156 (45.22) | 1.212 (0.884–1.661) | 0.233 | 1.311 (0.946–1.817) | 0.104 |  |
|  |  |  | TT | 43 (11.85) | 42 (12.17) | 1.075 (0.662–1.744) | 0.770 | 1.134 (0.685–1.875) | 0.625 |  |
|  |  | Additive | CC vs TC vs TT | |  | 1.087 (0.871–1.357) | 0.459 | 1.135 (0.903–1.428) | 0.278 | 959.623 |
|  |  | Dominant^*^ | CC | 140 (38.57) | 147 (42.61) | 1.0 (Ref.) | – | 1.0 (Ref.) | – | 958.475 |
|  |  |  | TC + TT | 223 (61.43) | 198 (57.39) | 1.183 (0.876–1.597) | 0.274 | 1.273 (0.933–1.737) | 0.128 |  |
|  |  | Recessive | CC + TC | 320 (88.15) | 303 (87.83) | 1.0 (Ref.) | – | 1.0 (Ref.) | – | 960.797 |
|  |  |  | TT | 43 (11.85) | 42 (12.17) | 0.969 (0.616–1.525) | 0.893 | 0.980 (0.612–1.569) | 0.933 |  |
|  | rs707265 | Codominant | GG | 126 (34.71) | 133 (38.55) | 1.0 (Ref.) | – | 1.0 (Ref.) | – | 959.628 |
|  |  |  | AG | 190 (52.34) | 164 (47.54) | 1.233 (0.887–1.686) | 0.219 | 1.339 (0.960–1.869) | 0.086 |  |
|  |  |  | AA | 47 (12.95) | 48 (13.91) | 1.034 (0.6646–1.654) | 0.891 | 1.068 (0.656–1.739) | 0.791 |  |
|  |  | Additive | GG vs AG vs AA | |  | 1.067 (0.855–1.330) | 0.567 | 1.105 (0.879–1.389) | 0.392 | 960.071 |
|  |  | Dominant^*^ | GG | 126 (34.71) | 133 (38.55) | 1.0 (Ref.) | – | 1.0 (Ref.) | – | 958.523 |
|  |  |  | AG + AA | 237 (65.29) | 212 (61.45) | 1.180 (0.869–1.603) | 0.289 | 1.277 (0.929–1.755) | 0.132 |  |
|  |  | Recessive | GG + AG | 316 (87.05) | 297 (86.09) | 1.0 (Ref.) | – | 1.0 (Ref.) | – | 960.596 |
|  |  |  | AA | 47 (12.95) | 48 (13.91) | 0.921 (0.597–1.418) | 0.706 | 0.901 (0.577–1.409) | 0.649 |  |
| *CYP2D6* | rs1135840 | Codominant | GG | 182 (50.14) | 181 (52.46) | 1.0 (Ref.) | – | 1.0 (Ref.) | – | 962.385 |
|  |  |  | GC | 125 (34.44) | 117 (33.91) | 1.063 (0.767–1.471) | 0.715 | 1.070 (0.765–1.496) | 0.694 |  |
|  |  |  | CC | 56 (15.43) | 47 (13.62) | 1.185 (0.764–1.838) | 0.449 | 1.151 (0.731–1.810) | 0.544 |  |
|  |  | Additive^*^ | GG vs GC vs CC | |  | 1.082 (0.883–1.327) | 0.448 | 1.072 (0.868–1.323) | 0.518 | 960.386 |
|  |  | Dominant | GG | 182 (50.14) | 181 (52.46) | 1.0 (Ref.) | – | 1.0 (Ref.) | – | 960.475 |
|  |  |  | GC + CC | 181 (49.86) | 164 (47.54) | 1.098 (0.817–1.474) | 0.536 | 1.093 (0.806–1.482) | 0.567 |  |
|  |  | Recessive | GG + GC | 307 (84.57) | 298 (86.38) | 1.0 (Ref.) | – | 1.0 (Ref.) | – | 960.540 |
|  |  |  | CC | 56 (15.43) | 47 (13.62) | 1.157 (0.761–1.759) | 0.496 | 1.120 (0.727–1.726) | 0.608 |  |
|  | rs16947 | Codominant | GG | 238 (65.56) | 220 (63.77) | 1.0 (Ref.) | – | 1.0 (Ref.) | – | 962.792 |
|  |  |  | GA | 122 (33.61) | 121 (35.07) | 0.932 (0.683–1.272) | 0.657 | 0.989 (0.716–1.367) | 0.947 |  |
|  |  |  | AA | 3 (0.83) | 4 (1.16) | 0.693 (0.153–3.132) | 0.634 | 0.930 (0.198–4.378) | 0.927 |  |
|  |  | Additive^*^ | GG vs GA vs AA | |  | 0.919 (0.685–1.233) | 0.572 | 0.986 (0.726–1.338) | 0.927 | 960.795 |
|  |  | Dominant | GG | 238 (65.56) | 220 (63.77) | 1.0 (Ref.) | – | 1.0 (Ref.) | – | 960.798 |
|  |  |  | GA + AA | 125 (34.44) | 125 (36.23) | 0.924 (0.679–1.258) | 0.617 | 0.987 (0.717–1.360) | 0.938 |  |
|  |  | Recessive | GG + GA | 360 (99.17) | 341 (98.84) | 1.0 (Ref.) | – | 1.0 (Ref.) | – | 960.796 |
|  |  |  | AA | 3 (0.83) | 4 (1.16) | 0.710 (0.158–3.197) | 0.656 | 0.934 (0.199–4.377) | 0.931 |  |
|  | rs1081003 | Codominant | GG | 98 (27.00) | 96 (27.83) | 1.0 (Ref.) | – | 1.0 (Ref.) | – | 962.575 |
|  |  |  | GA | 179 (49.31) | 164 (47.54) | 1.069 (0.752–1.521) | 0.710 | 0.967 (0.671–1.395) | 0.859 |  |
|  |  |  | AA | 86 (23.69) | 85 (24.64) | 0.991 (0.657–1.495) | 0.966 | 0.903 (0.589–1.384) | 0.639 |  |
|  |  | Additive^*^ | GG vs GA vs AA | |  | 0.998 (0.812–1.225) | 0.983 | 0.951 (0.768–1.177) | 0.642 | 960.588 |
|  |  | Dominant | GG | 98 (27.00) | 96 (27.83) | 1.0 (Ref.) | – | 1.0 (Ref.) | – | 960.701 |
|  |  |  | GA + AA | 265 (73.00) | 249 (72.17) | 1.043 (0.749–1.451) | 0.805 | 0.946 (0.671–1.333) | 0.749 |  |
|  |  | Recessive | GG + GA | 277 (76.31) | 260 (75.36) | 1.0 (Ref.) | – | 1.0 (Ref.) | – | 960.606 |
|  |  |  | AA | 86 (23.69) | 85 (24.64) | 0.950 (0.673–1.340) | 0.769 | 0.922 (0.646–1.317) | 0.657 |  |
|  | rs1065852 | Codominant | GG | 102 (28.10) | 92 (26.67) | 1.0 (Ref.) | – | 1.0 (Ref.) | – | 959.533 |
|  |  |  | GA | 183 (50.41) | 162 (46.96) | 1.019 (0.716–1.449) | 0.917 | 0.957 (0.664–1.380) | 0.815 |  |
|  |  |  | AA | 78 (21.49) | 91 (26.38) | 0.773 (0.511–1.169) | 0.222 | 0.700 (0.455–1.077) | 0.105 |  |
|  |  | Additive | GG vs GA vs AA | |  | 0.884 (0.719–1.086) | 0.240 | 0.840 (0.678–1.042) | 0.113 | 958.277 |
|  |  | Dominant | GG | 102 (28.10) | 92 (26.67) | 1.0 (Ref.) | – | 1.0 (Ref.) | – | 960.120 |
|  |  |  | GA + AA | 261 (71.90) | 253 (73.33) | 0.930 (0.669–1.295) | 0.669 | 0.865 (0.613–1.220) | 0.409 |  |
|  |  | Recessive^*^ | GG + GA | 285 (78.51) | 254 (73.62) | 1.0 (Ref.) | – | 1.0 (Ref.) | – | 957.588 |
|  |  |  | AA | 78 (21.49) | 91 (26.38) | 0.764 (0.540–1.080) | 0.128 | 0.720 (0.503–1.032) | 0.074 |  |
| *AOX1* | rs2072034 | Codominant | CC | 131 (36.09) | 103 (29.86) | 1.0 (Ref.) | – | 1.0 (Ref.) | – | 959.289 |
|  |  |  | GC | 179 (49.31) | 184 (53.33) | 0.765 (0.550–1.064) | 0.112 | 0.735 (0.523–1.033) | 0.076 |  |
|  |  |  | GG | 53 (14.60) | 58 (16.81) | 0.718 (0.457–1.130) | 0.153 | 0.730 (0.458–1.164) | 0.187 |  |
|  |  | Additive | CC vs GC vs GG | |  | 0.831 (0.668–1.034) | 0.097 | 0.830 (0.663–1.040) | 0.105 | 958.161 |
|  |  | Dominant^*^ | CC | 131 (36.09) | 103 (29.86) | 1.0 (Ref.) | – | 1.0 (Ref.) | – | 957.289 |
|  |  |  | GC + GG | 232 (63.91) | 242 (70.14) | 0.754 (0.550–1.033) | 0.078 | 0.734 (0.531–1.015) | 0.061 |  |
|  |  | Recessive | CC + GC | 310 (85.40) | 287 (83.19) | 1.0 (Ref.) | – | 1.0 (Ref.) | – | 960.411 |
|  |  |  | GG | 53 (14.60) | 58 (16.81) | 0.846 (0.564–1.269) | 0.419 | 0.880 (0.579–1.336) | 0.547 |  |
|  | rs10931910 | Codominant | GG | 274 (75.48) | 259 (75.07) | 1.0 (Ref.) | – | 1.0 (Ref.) | – | 960.495 |
|  |  |  | GA | 79 (21.26) | 82 (23.77) | 0.911 (0.640–1.296) | 0.603 | 0.943 (0.656–1.356) | 0.753 |  |
|  |  |  | AA | 10 (2.75) | 4 (1.16) | 2.363 (0.732–7.629) | 0.150 | 2.385 (0.708–8.037) | 0.161 |  |
|  |  | Additive | GG vs GA vs AA | |  | 1.052 (0.776–1.426) | 0.745 | 1.077 (0.787–1.474) | 0.643 | 960.589 |
|  |  | Dominant | GG | 274 (75.48) | 259 (75.07) | 1.0 (Ref.) | – | 1.0 (Ref.) | – | 960.801 |
|  |  |  | GA + AA | 89 (24.52) | 86 (24.93) | 0.978 (0.695–1.377) | 0.899 | 1.010 (0.711–1.436) | 0.955 |  |
|  |  | Recessive^*^ | GG + GA | 353 (98.02) | 341 (98.84) | 1.0 (Ref.) | – | 1.0 (Ref.) | – | 958.594 |
|  |  |  | AA | 10 (1.98) | 4 (1.16) | 2.415 (0.750–7.774) | 0.139 | 2.416 (0.719–8.121) | 0.154 |  |
|  | rs3731722 | Codominant | AA | 304 (83.75) | 294 (85.22) | 1.0 (Ref.) | – | 1.0 (Ref.) | – | 962.521 |
|  |  |  | AG | 54 (14.88) | 48 (13.91) | 1.088 (0.714–1.657) | 0.694 | 1.120 (0.726–1.727) | 0.609 |  |
|  |  |  | GG | 5 (1.38) | 3 (0.87) | 1.612 (0.382–6.805) | 0.516 | 1.130 (0.260–4.908) | 0.870 |  |
|  |  | Additive | AA vs AG vs GG | |  | 1.131 (0.783–1.634) | 0.513 | 1.105 (0.756–1.617) | 0.606 | 960.536 |
|  |  | Dominant^*^ | AA | 304 (83.75) | 294 (85.22) | 1.0 (Ref.) | – | 1.0 (Ref.) | – | 960.522 |
|  |  |  | AG + GG | 59 (16.25) | 51 (14.78) | 1.119 (0.744–1.682) | 0.589 | 1.120 (0.736–1.705) | 0.596 |  |
|  |  | Recessive | AA + AG | 358 (98.62) | 342 (99.13) | 1.0 (Ref.) | – | 1.0 (Ref.) | – | 960.783 |
|  |  |  | GG | 5 (1.38) | 3 (0.87) | 1.592 (0.378–6.713) | 0.526 | 1.113 (0.257–4.828) | 0.886 |  |
| *FMO3* | rs1736555 | Codominant | GG | 142 (39.12) | 123 (35.65) | 1.0 (Ref.) | – | 1.0 (Ref.) | – | 959.477 |
|  |  |  | GA | 170 (46.83) | 159 (46.09) | 0.926 (0.670–1.281) | 0.643 | 0.914 (0.654–1.278) | 0.600 |  |
|  |  |  | AA | 51 (14.05) | 63 (18.26) | 0.701 (0.451–1.090) | 0.115 | 0.657 (0.654–1.278) | 0.072 |  |
|  |  | Additive | GG vs GA vs AA | |  | 0.855 (0.692–1.056) | 0.145 | 0.831 (0.667–1.034) | 0.097 | 958.031 |
|  |  | Dominant | GG | 142 (39.12) | 123 (35.65) | 1.0 (Ref.) | – | 1.0 (Ref.) | – | 959.642 |
|  |  |  | GA + AA | 221 (60.88) | 222 (64.35) | 0.862 (0.636–1.170) | 0.341 | 0.841 (0.614–1.153) | 0.281 |  |
|  |  | Recessive^*^ | GG + GA | 312 (85.95) | 282 (81.74) | 1.0 (Ref.) | – | 1.0 (Ref.) | – | 957.753 |
|  |  |  | AA | 51 (14.05) | 63 (18.26) | 0.732 (0.489–1.095) | 0.128 | 0.690 (0.455–1.048) | 0.082 |  |
|  | rs10911192 | Codominant | AA | 98 (27.00) | 112 (32.46) | 1.0 (Ref.) | – | 1.0 (Ref.) | – | 958.668 |
|  |  |  | CA | 202 (55.65) | 170 (49.28) | 1.358 (0.967–1.907) | 0.077 | 1.422 (1.000–2.024) | 0.050 |  |
|  |  |  | CC | 63 (17.36) | 63 (18.26) | 1.143 (0.735–1.778) | 0.554 | 1.124 (0.709–1.779) | 0.619 |  |
|  |  | Additive | AA vs CA vs CC | |  | 1.104 (0.888–1.372) | 0.372 | 1.103 (0.880–1.383) | 0.396 | 960.081 |
|  |  | Dominant^*^ | AA | 98 (27.00) | 112 (32.46) | 1.0 (Ref.) | – | 1.0 (Ref.) | – | 957.879 |
|  |  |  | CA + CC | 265 (73.00) | 233 (67.54) | 1.300 (0.941–1.796) | 0.112 | 1.340 (0.958–1.875) | 0.088 |  |
|  |  | Recessive | AA + CA | 300 (82.64) | 282 (81.74) | 1.0 (Ref.) | – | 1.0 (Ref.) | – | 960.517 |
|  |  |  | CC | 63 (17.36) | 63 (18.26) | 0.940 (0.639–1.382) | 0.753 | 0.897 (0.601–1.338) | 0.593 |  |
|  | rs2266782 | Codominant | GG | 221 (60.88) | 228 (66.09) | 1.0 (Ref.) | – | 1.0 (Ref.) | – | 960.845 |
|  |  |  | AG | 121 (33.33) | 101 (29.28) | 1.236 (0.895–1.706) | 0.198 | 1.246 (0.891–1.742) | 0.199 |  |
|  |  |  | AA | 21 (5.79) | 16 (4.64) | 1.354 (0.689–2.663) | 0.380 | 1.302 (0.648–2.614) | 0.459 |  |
|  |  | Additive | GG vs AG vs AA | |  | 1.201 (0.934–1.545) | 0.153 | 1.195 (0.921–1.549) | 0.180 | 958.993 |
|  |  | Dominant^*^ | GG | 221 (60.88) | 228 (66.09) | 1.0 (Ref.) | – | 1.0 (Ref.) | – | 958.859 |
|  |  |  | AG + AA | 142 (39.12) | 117 (33.91) | 1.252 (0.921–1.702) | 0.151 | 1.254 (0.912–1.723) | 0.164 |  |
|  |  | Recessive | GG + AG | 342 (94.21) | 329 (95.36) | 1.0 (Ref.) | – | 1.0 (Ref.) | – | 960.497 |
|  |  |  | AA | 21 (5.79) | 16 (4.64) | 1.263 (0.648–2.462) | 0.494 | 1.214 (0.610–2.420) | 0.581 |  |
|  | rs1736557 | Codominant | GG | 224 (61.71) | 216 (62.61) | 1.0 (Ref.) | – | 1.0 (Ref.) | – | 961.225 |
|  |  |  | GA | 126 (34.71) | 112 (32.46) | 1.085 (0.791–1.488) | 0.613 | 1.154 (0.832–1.600) | 0.392 |  |
|  |  |  | AA | 13 (3.58) | 17 (4.93) | 0.737 (0.350–1.555) | 0.423 | 0.737 (0.342–1.590) | 0.436 |  |
|  |  | Additive | GG vs GA vs AA | |  | 0.987 (0.763–1.276) | 0.918 | 1.022 (0.784–1.333) | 0.871 | 960.777 |
|  |  | Dominant | GG | 224 (61.71) | 216 (62.61) | 1.0 (Ref.) | – | 1.0 (Ref.) | – | 960.473 |
|  |  |  | GA + AA | 139 (38.29) | 129 (37.39) | 1.039 (0.767–1.408) | 0.805 | 1.097 (0.801–1.502) | 0.566 |  |
|  |  | Recessive^*^ | GG + GA | 350 (96.42) | 328 (95.07) | 1.0 (Ref.) | – | 1.0 (Ref.) | – | 959.960 |
|  |  |  | AA | 13 (3.58) | 17 (4.93) | 0.717 (0.343–1.499) | 0.376 | 0.701 (0.328–1.500) | 0.360 |  |
|  | rs2075992 | Codominant | TT | 155 ( (42.70) | 141 (40.87) | 1.0 (Ref.) | – | 1.0 (Ref.) | – | 961.053 |
|  |  |  | TC | 163 (44.90) | 153 (44.35) | 0.969 (0.706–1.331) | 0.846 | 0.953 (0.687–1.322) | 0.772 |  |
|  |  |  | CC | 45 (12.40) | 51 (14.78) | 0.803 (0.506–1.273) | 0.350 | 0.726 (0.449–1.174) | 0.191 |  |
|  |  | Additive | TT vs TC vs CC | |  | 0.915 (0.738–1.133) | 0.415 | 0.878 (0.703–1.097) | 0.253 | 959.495 |
|  |  | Dominant | TT | 155 (42.70) | 141 (40.87) | 1.0 (Ref.) | – | 1.0 (Ref.) | – | 960.316 |
|  |  |  | TC + CC | 208 (57.30) | 204 (59.13) | 0.928 (0.688–1.251) | 0.622 | 0.896 (0.657–1.220) | 0.485 |  |
|  |  | Recessive^*^ | TT + TC | 318 (87.60) | 294 (85.22) | 1.0 (Ref.) | – | 1.0 (Ref.) | – | 959.137 |
|  |  |  | CC | 45 (12.40) | 51 (14.78) | 0.816 (0.530–1.255) | 0.355 | 0.744 (0.475–1.166) | 0.198 |  |
|  | rs909529 | Codominant | CC | 211 (58.13) | 215 (62.32) | 1.0 (Ref.) | – | 1.0 (Ref.) | – | 961.187 |
|  |  |  | CT | 132 (36.36) | 111 (32.17) | 1.212 (0.884–1.662) | 0.233 | 1.236 (0.891–1.714) | 0.205 |  |
|  |  |  | TT | 20 (5.51) | 19 (5.51) | 1.073 (0.557–2.067) | 0.834 | 1.049 (0.530–2.076) | 0.892 |  |
|  |  | Additive | CC vs CT vs TT | |  | 1.125 (0.878–1.440) | 0.351 | 1.131 (0.875–1.461) | 0.347 | 959.918 |
|  |  | Dominant^*^ | CC | 211 (58.13) | 215 (62.32) | 1.0 (Ref.) | – | 1.0 (Ref.) | – | 959.395 |
|  |  |  | CT + TT | 152 (41.87) | 130 (37.68) | 1.191 (0.881–1.611) | 0.255 | 1.208 (0.884–1.651) | 0.236 |  |
|  |  | Recessive | CC + CT | 342 (94.49) | 326 (94.49) | 1.0 (Ref.) | – | 1.0 (Ref.) | – | 960.797 |
|  |  |  | TT | 20 (5.51) | 19 (5.51) | 1.000 (0.524–1.909) | 0.999 | 0.972 (0.496–1.904) | 0.933 |  |
|  | rs909530 | Codominant | CC | 125 (34.44) | 135 (39.13) | 1.0 (Ref.) | – | 1.0 (Ref.) | – | 960.362 |
|  |  |  | TC | 185 (50.96) | 162 (49.96) | 1.233 (0.894–1.702) | 0.202 | 1.302 (0.933–1.818) | 0.120 |  |
|  |  |  | TT | 53 (4.60) | 48 (13.91) | 1.193 (0.753–1.889) | 0.453 | 1.125 (0.698–1.814) | 0.629 |  |
|  |  | Additive | CC vs TC vs TT | |  | 1.124 (0.904–1.398) | 0.291 | 1.115 (0.889–1.398) | 0.345 | 959.912 |
|  |  | Dominant^*^ | CC | 125 (34.44) | 135 (39.13) | 1.0 (Ref.) | – | 1.0 (Ref.) | – | 958.750 |
|  |  |  | TC + TT | 238 (65.56) | 210 (60.87) | 1.224 (0.901–1.662) | 0.195 | 1.260 (0.918–1.730) | 0.152 |  |
|  |  | Recessive | CC + TC | 310 (85.40) | 297 (86.09) | 1.0 (Ref.) | – | 1.0 (Ref.) | – | 960.781 |
|  |  |  | TT | 53 (14.60) | 48 (13.91) | 1.058 (0.694–1.613) | 0.794 | 0.967 (0.624–1.498) | 0.880 |  |
|  | rs2266780 | Codominant | AA | 238 (65.56) | 234 (67.83) | 1.0 (Ref.) | – | 1.0 (Ref.) | – | 962.496 |
|  |  |  | GA | 111 (30.58) | 97 (28.12) | 1.125 (0.811–1.560) | 0.480 | 1.084 (0.771–1.524) | 0.643 |  |
|  |  |  | GG | 14 (3.86) | 14 (4.06) | 0.983 (0.459–2.108) | 0.965 | 0.908 (0.414–1.990) | 0.810 |  |
|  |  | Additive | AA vs GA vs GG | |  | 1.068 (0.821–1.390) | 0.624 | 1.027 (0.781–1.350) | 0.849 | 960.767 |
|  |  | Dominant^*^ | AA | 238 (65.56) | 234 (67.83) | 1.0 (Ref.) | – | 1.0 (Ref.) | – | 960.678 |
|  |  |  | GA + GG | 125 (34.44) | 111 (32.17) | 1.107 (0.810–1.514) | 0.524 | 1.061 (0.766–1.469) | 0.723 |  |
|  |  | Recessive | AA + GA | 349 (96.14) | 331 (95.94) | 1.0 (Ref.) | – | 1.0 (Ref.) | – | 960.711 |
|  |  |  | GG | 14 (3.86) | 14 (4.06) | 0.948 (0.445–2.020) | 0.891 | 0.886 (0.407–1.929) | 0.761 |  |
|  | rs909531 | Codominant | TT | 236 (65.01) | 233 (67.54) | 1.0 (Ref.) | – | 1.0 (Ref.) | – | 962.301 |
|  |  |  | TC | 112 (30.85) | 96 (27.83) | 1.152 (0.830–1.598) | 0.397 | 1.107 (0.787–1.557) | 0.558 |  |
|  |  |  | CC | 15 (4.13) | 16 (4.64) | 0.926 (0.447–1.915) | 0.835 | 0.887 (0.419–1.877) | 0.755 |  |
|  |  | Additive | TT vs TC vs CC | |  | 1.064 (0.821–1.380) | 0.637 | 1.030 (0.787–1.349) | 0.828 | 960.756 |
|  |  | Dominant^*^ | TT | 236 (65.01) | 233 (67.54) | 1.0 (Ref.) | – | 1.0 (Ref.) | – | 960.612 |
|  |  |  | TC + CC | 127 (34.99) | 112 (32.46) | 1.120 (0.820–1.529) | 0.478 | 1.075 (0.777–1.488) | 0.662 |  |
|  |  | Recessive | TT + TC | 348 (95.87) | 329 (95.36) | 1.0 (Ref.) | – | 1.0 (Ref.) | – | 960.644 |
|  |  |  | CC | 15 (4.13) | 16 (4.64) | 0.886 (0.431–1.822) | 0.743 | 0.860 (0.409–1.805) | 0.690 |  |
| *UGT1A4* | rs3806594 | Codominant | TT | 249 (68.60) | 221 (64.06) | 1.0 (Ref.) | – | 1.0 (Ref.) | – | 960.360 |
|  |  |  | TC | 102 (28.10) | 116 (33.62) | 0.780 (0.566–1.077) | 0.131 | 0.799 (0.572–1.115) | 0.186 |  |
|  |  |  | CC | 12 (3.31) | 8 (2.32) | 1.331 (0.534–3.317) | 0.539 |  |  |  |
|  |  | Additive | TT vs TC vs CC | |  | 0.884 (0.672–1.164) | 0.379 |  |  | 960.326 |
|  |  | Dominant^*^ | TT | 249 (68.60) | 221 (64.06) | 1.0 (Ref.) | – | 1.0 (Ref.) | – | 959.640 |
|  |  |  | TC + CC | 114 (31.40) | 124 (35.94) | 0.816 (0.597–1.115) | 0.202 |  |  |  |
|  |  | Recessive | TT + TC | 351 (96.69) | 337 (97.68) | 1.0 (Ref.) | – | 1.0 (Ref.) | – | 960.113 |
|  |  |  | CC | 12 (3.31) | 8 (2.32) | 1.441 (0.581–3.567) | 0.431 |  |  |  |
|  | rs3732217 | Codominant | GG | 252 (69.42) | 221 (64.06) | 1.0 (Ref.) | – | 1.0 (Ref.) | – | 960.321 |
|  |  |  | GA | 101 (27.82) | 116 (33.62) | 0.764 (0.553–1.054) | 0.101 | 0.779 (0.559–1.088) | 0.143 |  |
|  |  |  | AA | 10 (2.75) | 8 (2.32) | 1.096 (0.425–2.826) | 0.849 | 1.229 (0.464–3.256) | 0.679 |  |
|  |  | Additive | GG vs GA vs AA | |  | 0.839 (0.635–1.108) | 0.216 | 0.866 (0.649–1.155) | 0.328 | 959.845 |
|  |  | Dominant^*^ | GG | 252 (69.42) | 221 (64.06) | 1.0 (Ref.) | – | 1.0 (Ref.) | – | 959.128 |
|  |  |  | GA + AA | 111 (30.58) | 124 (35.94) | 0.785 (0.574–1.074) | 0.130 | 0.808 (0.584–1.116) | 0.196 |  |
|  |  | Recessive | GG + GA | 353 (97.25) | 337 (97.68) | 1.0 (Ref.) | – | 1.0 (Ref.) | – | 960.473 |
|  |  |  | AA | 10 (2.75) | 8 (2.32) | 1.193 (0.465–3.060) | 0.713 | 1.328 (0.504–3.500) | 0.566 |  |
| *UGT2B10* | rs2942857 | Codominant | AA | 298 (82.09) | 277 (80.29) | 1.0 (Ref.) | – | 1.0 (Ref.) | – | 960.535 |
|  |  |  | CA | 65 (17.91) | 68 (19.71) | 0.889 (0.609–1.296) | 0.539 | 0.902 (0.610–1.333) | 0.604 |  |
|  |  |  | CC | 0 | 0 |  |  |  |  |  |
|  | rs11726322 | Codominant | GG | 245 (67.49) | 246 (71.30) | 1.0 (Ref.) | – | 1.0 (Ref.) | – | 957.389 |
|  |  |  | CG | 103 (28.37) | 94 (27.25) | 1.100 (0.790–1.532) | 0.572 | 1.111 (0.790–1.563) | 0.544 |  |
|  |  |  | CC | 15 (4.13) | 5 (1.45) | 3.012 (1.078–8.416) | 0.035 | 3.189 (1.112–9.151) | 0.031 |  |
|  |  | Additive | GG vs CG vs CC | |  | 1.264 (0.954–1.674) | 0.103 | 1.283 (0.960–1.716) | 0.092 | 957.949 |
|  |  | Dominant | GG | 245 (67.49) | 246 (71.30) | 1.0 (Ref.) | – | 1.0 (Ref.) | – | 959.491 |
|  |  |  | CG + CC | 118 (32.51) | 99 (28.70) | 1.197 (0.869–1.649) | 0.272 | 1.213 (0.872–1.687) | 0.252 |  |
|  |  | Recessive^*^ | GG + CG | 348 (95.87) | 340 (98.55) | 1.0 (Ref.) | – | 1.0 (Ref.) | – | 955.758 |
|  |  |  | CC | 15 (4.13) | 5 (1.45) | 2.931 (1.053–8.154) | 0.039 | 3.094 (1.083–8.837) | 0.035 |  |
|  | rs4694358 | Codominant | TT | 265 (73.00) | 262 (75.94) | 1.0 (Ref.) | – | 1.0 (Ref.) | – | 959.818 |
|  |  |  | TC | 98 (27.00) | 83 (24.06) | 1.167 (0.832–1.638) | 0.370 | 1.193 (0.841–1.692) | 0.321 |  |
|  |  |  | CC | 0 | 0 |  |  |  |  |  |
| *UGT1A9* | rs17864684 | Codominant | GG | 274 (75.48) | 248 (71.88) | 1.0 (Ref.) | – | 1.0 (Ref.) | – | 959.420 |
|  |  |  | AG | 82 (22.59) | 93 (26.96) | 0.798 (0.566–1.125) | 0.197 | 0.749 (0.523–1.072) | 0.114 |  |
|  |  |  | AA | 7 (1.93) | 4 (1.16) | 1.584 (0.458–5.476) | 0.467 | 1.686 (0.476–5.969) | 0.418 |  |
|  |  | Additive | GG vs AG vs AA | |  | 0.885 (0.652–1.202) | 0.435 | 0.854 (0.621–1.172) | 0.328 | 959.844 |
|  |  | Dominant^*^ | GG | 274 (75.48) | 248 (71.88) | 1.0 (Ref.) | – | 1.0 (Ref.) | – | 959.002 |
|  |  |  | AG + AA | 89 (24.52) | 97 (28.12) | 0.830 (0.594–1.161) | 0.277 | 0.788 (0.556–1.117) | 0.180 |  |
|  |  | Recessive | GG + AG | 356 (98.07) | 341 (98.84) | 1.0 (Ref.) | – | 1.0 (Ref.) | – | 959.933 |
|  |  |  | AA | 7 (1.93) | 4 (1.16) | 1.676 (0.486–5.778) | 0.413 | 1.803 (0.511–6.364) | 0.360 |  |
|  | rs7349250 | Codominant | AA | 217 (59.78) | 207 (60.00) | 1.0 (Ref.) | – | 1.0 (Ref.) | – | 960.885 |
|  |  |  | GA | 124 (34.16) | 126 (36.52) | 0.939 (0.687–1.283) | 0.692 | 0.969 (0.701–1.339) | 0.847 |  |
|  |  |  | GG | 22 (6.06) | 12 (3.48) | 1.749 (0.844–3.624) | 0.133 | 1.645 (0.778–3.477) | 0.193 |  |
|  |  | Additive | AA vs GA vs GG | |  | 1.085 (0.844–1.396) | 0.525 | 1.091 (0.841–1.415) | 0.511 | 960.372 |
|  |  | Dominant | AA | 217 (59.78) | 207 (60.00) | 1.0 (Ref.) | – | 1.0 (Ref.) | – | 960.767 |
|  |  |  | GA + GG | 146 (40.22) | 138 (40.00) | 1.010 (0.747–1.363) | 0.952 | 1.031 (0.756–1.407) | 0.847 |  |
|  |  | Recessive^*^ | AA + GA | 341 (93.94) | 333 (96.52) | 1.0 (Ref.) | – | 1.0 (Ref.) | – | 958..923 |
|  |  |  | GG | 22 (6.06) | 12 (3.48) | 1.790 (0.872–3.676) | 0.113 | 1.664 (0.794–3.485) | 0.177 |  |
|  | rs2602379 | Codominant | GG | 106 (29.20) | 106 (30.72) | 1.0 (Ref.) | – | 1.0 (Ref.) | – | 961.214 |
|  |  |  | GA | 177 (48.76) | 176 (51.01) | 1.006 (0.715–1.414) | 0.974 | 1.016 (0.714–1.444) | 0.930 |  |
|  |  |  | AA | 80 (22.04) | 63 (18.26) | 1.270 (0.829–1.944) | 0.272 | 1.289 (0.830–2.001) | 0.258 |  |
|  |  | Additive | GG vs GA vs AA | |  | 1.114 (0.903–1.375) | 0.315 | 1.123 (0.903–1.395) | 0.297 | 959.714 |
|  |  | Dominant | GG | 106 (29.20) | 106 (30.72) | 1.0 (Ref.) | – | 1.0 (Ref.) | – | 960.556 |
|  |  |  | GA + AA | 257 (70.80) | 239 (69.28) | 1.075 (0.779–1.483) | 0.658 | 1.088 (0.780–1.517) | 0.619 |  |
|  |  | Recessive^*^ | GG + GA | 283 (77.96) | 282 (81.74) | 1.0 (Ref.) | – | 1.0 (Ref.) | – | 959.222 |
|  |  |  | AA | 80 (22.04) | 63 (18.26) | 1.265 (0.875–1.830) | 0.211 | 1.276 (0.872–1.869) | 0.209 |  |
|  | rs1604144 | Codominant | CC | 211 (58.13) | 190 (55.07) | 1.0 (Ref.) | – | 1.0 (Ref.) | – | 962.186 |
|  |  |  | CT | 131 (36.09) | 135 (39.13) | 0.874 (0.641–1.192) | 0.394 | 0.881 (0.639–1.215) | 0.441 |  |
|  |  |  | TT | 21 (5.79) | 20 (5.80) | 0.945 (0.497–1.798) | 0.864 | 0.902 (0.462–1.763) | 0.763 |  |
|  |  | Additive | CC vs CT vs TT | |  | 0.920 (0.721–1.173) | 0.500 | 0.913 (0.709–1.177) | 0.483 | 960.311 |
|  |  | Dominant^*^ | CC | 211 (58.13) | 190 (55.07) | 1.0 (Ref.) | – | 1.0 (Ref.) | – | 960.191 |
|  |  |  | CT + TT | 152 (41.87) | 155 (44.93) | 0.883 (0.656–1.189) | 0.412 | 0.884 (0.649–1.204) | 0.434 |  |
|  |  | Recessive | CC + CT | 342 (94.21) | 325 (94.20) | 1.0 (Ref.) | – | 1.0 (Ref.) | – | 960.780 |
|  |  |  | TT | 21 (5.79) | 20 (5.80) | 0.998 (0.531–1.875) | 0.995 | 0.950 (0.493–1.832) | 0.878 |  |
|  | rs12988520 | Codominant | AA | 194 (53.44) | 190 (55.07) | 1.0 (Ref.) | – | 1.0 (Ref.) | – | 962.582 |
|  |  |  | CA | 136 (37.47) | 125 (36.23) | 1.066 (0.778–1.460) | 0.692 | 1.051 (0.758–1.457) | 0.767 |  |
|  |  |  | CC | 33 (9.09) | 30 (8.70) | 1.077 (0.632–1.836) | 0.784 | 1.127 (0.650–1.196) | 0.670 |  |
|  |  | Additive^*^ | AA vs CA vs CC | |  | 1.049 (0.837–1.315) | 0.680 | 1.057 (0.837–1.336) | 0.640 | 960.585 |
|  |  | Dominant | AA | 194 (53.44) | 190 (55.07) | 1.0 (Ref.) | – | 1.0 (Ref.) | – | 960.641 |
|  |  |  | CA + CC | 169 (46.56) | 155 (44.93) | 1.068 (0.794–1.435) | 0.664 | 1.065 (0.784–1.448) | 0.686 |  |
|  |  | Recessive | AA + CA | 330 (90.91) | 315 (91.30) | 1.0 (Ref.) | – | 1.0 (Ref.) | – | 960.670 |
|  |  |  | CC | 33 (9.09) | 30 (8.70) | 1.050 (0.626–1.762) | 0.854 | 1.105 (0.647–1.886) | 0.715 |  |
|  | rs2885295 | Codominant | TT | 248 (68.32) | 222 (64.35) | 1.0 (Ref.) | – | 1.0 (Ref.) | – | 958.857 |
|  |  |  | TA | 103 (28.37) | 117 (33.91) | 0.788 (0.572–1.086) | 0.146 | 0.772 (0.554–1.076) | 0.127 |  |
|  |  |  | AA | 12 (3.31) | 6 (1.74) | 1.790 (0.661–4.850) | 0.252 | 1.757 (0.633–4.875) | 0.279 |  |
|  |  | Additive | TT vs TA vs AA | |  | 0.918 (0.696–1.212) | 0.547 | 0.904 (0.679–1.204) | 0.492 | 960.331 |
|  |  | Dominant | TT | 248 (68.32) | 222 (64.35) | 1.0 (Ref.) | – | 1.0 (Ref.) | – | 959.374 |
|  |  |  | TA + AA | 115 (31.68) | 123 (35.65) | 0.837 (0.613–1.144) | 0.264 | 0.821 (0.595–1.134) | 0.232 |  |
|  |  | Recessive^*^ | TT + TA | 351 (96.69) | 339 (98.26) | 1.0 (Ref.) | – | 1.0 (Ref.) | – | 959.184 |
|  |  |  | AA | 12 (3.31) | 6 (1.74) | 1.932 (0.717–5.205) | 0.193 | 1.903 (0.690–5.252) | 0.214 |  |
|  | rs871514 | Codominant | TT | 246 (67.77) | 255 (73.91) | 1.0 (Ref.) | – | 1.0 (Ref.) | – | 958.861 |
|  |  |  | TC | 89 (24.52) | 65 (18.84) | 1.419 (0.986–2.044) | 0.060 | 1.453 (0.995–2.122) | 0.053 |  |
|  |  |  | CC | 28 (7.71) | 25 (7.25) | 1.161 (0.659–2.047) | 0.606 | 1.233 (0.683–2.226) | 0.487 |  |
|  |  | Additive | TT vs TC vs CC | |  | 1.190 (0.936–1.513) | 0.156 | 1.224 (0.953–1.571) | 0.113 | 958.271 |
|  |  | Dominant^*^ | TT | 246 (67.77) | 255 (73.91) | 1.0 (Ref.) | – | 1.0 (Ref.) | – | 957.104 |
|  |  |  | TC + CC | 117 (32.23) | 90 (26.09) | 1.348 (0.973–1.867) | 0.073 | 1.393 (0.993–1.955) | 0.055 |  |
|  |  | Recessive | TT + TC | 335 (92.29) | 320 (92.75) | 1.0 (Ref.) | – | 1.0 (Ref.) | – | 960.635 |
|  |  |  | CC | 28 (7.71) | 25 (7.25) | 1.070 (0.611–1.874) | 0.813 | 1.130 (0.630–2.027) | 0.681 |  |
|  | rs10178992 | Codominant | TT | 259 (71.35) | 263 (76.23) | 1.0 (Ref.) | – | 1.0 (Ref.) | – | 960.258 |
|  |  |  | AT | 99 (27.27) | 78 (22.61) | 1.289 (0.915–1.816) | 0.147 | 1.322 (0.926–1.888) | 0.124 |  |
|  |  |  | AA | 5 (1.38) | 4 (1.16) | 1.269 (0.337–4.780) | 0.724 | 1.428 (0.360–5.660) | 0.612 |  |
|  |  | Additive | TT vs AT vs AA | |  | 1.256 (0.919–1.717) | 0.153 | 1.296 (0.937–1.793) | 0.117 | 958.327 |
|  |  | Dominant^*^ | TT | 259 (71.35) | 263 (76.23) | 1.0 (Ref.) | – | 1.0 (Ref.) | – | 958.270 |
|  |  |  | AT + AA | 104 (28.65) | 82 (23.77) | 1.288 (0.920–1.803) | 0.141 | 1.327 (0.936–1.882) | 0.112 |  |
|  |  | Recessive | TT + AT | 358 (98.62) | 341 (98.84) | 1.0 (Ref.) | – | 1.0 (Ref.) | – | 960.630 |
|  |  |  | AA | 5 (1.38) | 4 (1.16) | 1.191 (0.317–4.471) | 0.796 | 1.338 (0.338–5.293) | 0.678 |  |
|  | rs10929303 | Codominant | CC | 263 (72.45) | 259 (75.07) | 1.0 (Ref.) | – | 1.0 (Ref.) | – | 962.127 |
|  |  |  | CT | 95 (26.17) | 81 (23.48) | 1.155 (0.820–1.627) | 0.410 | 1.132 (0.794–1.613) | 0.493 |  |
|  |  |  | TT | 5 (1.38) | 5 (1.45) | 0.985 (0.282–3.442) | 0.981 | 0.761 (0.203–2.853) | 0.686 |  |
|  |  | Additive | CC vs CT vs TT | |  | 1.118 (0.821–1.523) | 0.478 | 1.073 (0.778–1.481) | 0.666 | 960.617 |
|  |  | Dominant^*^ | CC | 263 (72.45) | 259 (75.07) | 1.0 (Ref.) | – | 1.0 (Ref.) | – | 960.460 |
|  |  |  | CT + TT | 100 (27.55) | 86 (24.93) | 0.145 (0.819–1.601) | 0.429 | 1.109 (0.784–1.570) | 0.558 |  |
|  |  | Recessive | CC + CT | 358 (98.62) | 340 (98.55) | 1.0 (Ref.) | – | 1.0 (Ref.) | – | 960.597 |
|  |  |  | TT | 5 (1.38) | 5 (1.45) | 0.950 (0.273–3.310) | 0.935 | 0.736 (0.197–2.751) | 0.649 |  |
| *UGT2B7* | rs12233719 | Codominant | GG | 245 (67.49) | 251 (72.75) | 1.0 (Ref.) | – | 1.0 (Ref.) | – | 955.477 |
|  |  |  | GT | 109 (30.03) | 93 (26.96) | 1.201 (0.865–1.667) | 0.274 | 1.184 (0.843–1.662) | 0.330 |  |
|  |  |  | TT | 9 (2.48) | 1 (0.29) | 9.220 (1.159–73.324) | 0.036 | 8.980 (1.086–72.632) | 0.042 |  |
|  |  | Additive | GG vs GT vs TT | |  | 1.361 (1.006–1.841) | 0.045 | 1.342 (0.982–1.834) | 0.065 | 957.374 |
|  |  | Dominant | GG | 245 (67.49) | 251 (72.75) | 1.0 (Ref.) | – | 1.0 (Ref.) | – | 958.856 |
|  |  |  | GT + TT | 118 (32.51) | 94 (27.25) | 1.286 (0.931–1.777) | 0.127 | 1.268 (0.908–1.771) | 0.164 |  |
|  |  | Recessive^*^ | GG + GT | 354 (97.52) | 334 (99.71) | 1.0 (Ref.) | – | 1.0 (Ref.) | – | 954.429 |
|  |  |  | TT | 9 (2.48) | 1 (0.29) | 8.746 (1.102–69.399) | 0.040 | 8.487 (1.040–69.282) | 0.046 |  |
|  | rs7439366 | Codominant | CC | 144 (39.67) | 132 (38.26) | 1.0 (Ref.) | – | 1.0 (Ref.) | – | 961.631 |
|  |  |  | TC | 175 (48.21) | 161 (46.67) | 0.996 (0.724–1.370) | 0.982 | 0.991 (0.713–1.378) | 0.959 |  |
|  |  |  | TT | 44 (12.12) | 52 (15.07) | 0.776 (0.487–1.236) | 0.285 | 0.777 (0.479–1.261) | 0.307 |  |
|  |  | Additive | CC vs TC vs TT | |  | 0.910 (0.732–1.130) | 0.393 | 0.909 (0.726–1.139) | 0.409 | 960.121 |
|  |  | Dominant | CC | 144 (39.67) | 132 (38.26) | 1.0 (Ref.) | – | 1.0 (Ref.) | – | 960.651 |
|  |  |  | TC + TT | 219 (60.33) | 213 (61.74) | 0.942 (0.697–1.275) | 0.701 | 0.940 (0.687–1.284) | 0.696 |  |
|  |  | Recessive^*^ | CC + TC | 319 (87.88) | 293 (84.93) | 1.0 (Ref.) | – | 1.0 (Ref.) | – | 959.634 |
|  |  |  | TT | 44 (12.12) | 52 (15.07) | 0.777 (0.505–1.197) | 0.252 | 0.781 (0.499–1.233) | 0.280 |  |
|  | rs12512526 | Codominant | CC | 144 (39.67) | 128 (37.10) | 1.0 (Ref.) | – | 1.0 (Ref.) | – | 961.278 |
|  |  |  | CT | 177 (48.76) | 168 (48.10) | 0.937 (0.681–1.287) | 0.686 | 0.913 (0.656–1.271) | 0.592 |  |
|  |  |  | TT | 42 (11.51) | 49 (14.20) | 0.762 (0.473–1.226) | 0.263 | 0.733 (0.488–1.202) | 0.218 |  |
|  |  | Additive^*^ | CC vs CT vs TT | |  | 0.890 (0.714–1.110) | 0.301 | 0.872 (0.693–1.096) | 0.241 | 959.425 |
|  |  | Dominant | CC | 144 (39.67) | 128 (37.10) | 1.0 (Ref.) | – | 1.0 (Ref.) | – | 960.086 |
|  |  |  | CT + TT | 219 (60.33) | 217 (62.90) | 0.897 (0.662–1.215) | 0.483 | 0.873 (0.637–1.196) | 0.397 |  |
|  |  | Recessive | CC + CT | 321 (88.43) | 296 (85.80) | 1.0 (Ref.) | – | 1.0 (Ref.) | – | 959.566 |
|  |  |  | TT | 42 (11.57) | 49 (14.20) | 0.790 (0.508–1.229) | 0.296 | 0.772 (0.489–1.219) | 0.267 |  |
|  | rs4292394 | Codominant | GG | 143 (39.39) | 126 (36.52) | 1.0 (Ref.) | – | 1.0 (Ref.) | – | 961.256 |
|  |  |  | CG | 178 (49.04) | 170 (49.28) | 0.923 (0.617–1.269) | 0.620 | 0.905 (0.650–1.261) | 0.557 |  |
|  |  |  | CC | 42 (11.57) | 49 (14.20) | 0.755 (0.469–1.216) | 0.248 | 0.732 (0.446–1.201) | 0.217 |  |
|  |  | Additive^*^ | GG vs CG vs CC | |  | 0.884 (0.708–1.102) | 0.273 | 0.869 (0.691–1.094) | 0.232 | 959.369 |
|  |  | Dominant | GG | 143 (39.39) | 126 (36.52) | 1.0 (Ref.) | – | 1.0 (Ref.) | – | 960.014 |
|  |  |  | CG + CC | 220 (61.61) | 219 (63.48) | 0.885 (0.653–1.119) | 0.431 | 0.866 (0.632–1.189) | 0.375 |  |
|  |  | Recessive | GG + CG | 321 (88.43) | 296 (85.80) | 1.0 (Ref.) | – | 1.0 (Ref.) | – | 959.601 |
|  |  |  | CC | 42 (11.57) | 49 (14.20) | 0.790 (0.508–1.229) | 0.296 | 0.775 (0.490–1.224) | 0.274 |  |
| *UGT2B15* | rs3100 | Codominant | GG | 237 (65.29) | 254 (73.62) | 1.0 (Ref.) | – | 1.0 (Ref.) | – | 953.359 |
|  |  |  | GA | 105 (28.93) | 83 (24.06) | 1.356 (0.967–1.901) | 0.077 | 1.389 (0.981–1.967) | 0.064 |  |
|  |  |  | AA | 21 (5.79) | 8 (2.32) | 2.813 (1.223–6.473) | 0.015 | 3.027 (1.281–7.154) | 0.012 |  |
|  |  | Additive^*^ | GG vs GA vs AA | |  | 1.476 (1.124–1.937) | 0.005 | 1.519 (1.147–2.012) | 0.004 | 952.090 |
|  |  | Dominant | GG | 237 (65.29) | 254 (73.62) | 1.0 (Ref.) |  | 1.0 (Ref.) | – | 954.508 |
|  |  |  | GA + AA | 126 (34.71) | 91 (26.38) | 1.484 (1.075–2.049) | 0.016 | 1.527 (1.095–2.130) | 0.013 |  |
|  |  | Recessive | GG + GA | 342 (94.21) | 337 (97.68) | 1.0 (Ref.) |  | 1.0 (Ref.) | – | 954.801 |
|  |  |  | AA | 21 (5.79) | 8 (2.32) | 2.587 (1.130–5.921) | 0.024 | 2.762 (1.175–6.489) | 0.020 |  |
|  | rs4148269 | Codominant | TT | 243 (66.94) | 242 (70.14) | 1.0 (Ref.) | – | 1.0 (Ref.) | – | 958.729 |
|  |  |  | GT | 99 (27.27) | 92 (26.67) | 1.072 (0.767–1.498) | 0.686 | 1.125 (0.795–1.592) | 0.506 |  |
|  |  |  | GG | 21 (5.79) | 11 (3.19) | 1.901 (0.897–4.028) | 0.094 | 2.145 (0.988–4.655) | 0.054 |  |
|  |  | Additive | TT vs GT vs GG | |  | 1.199 (0.923–1.558) | 0.174 | 1.266 (0.965–1.661) | 0.088 | 957.876 |
|  |  | Dominant | TT | 243 (66.94) | 242 (70.14) | 1.0 (Ref.) | – | 1.0 (Ref.) | – | 959.271 |
|  |  |  | GT + GG | 120 (33.06) | 103 (29.86) | 1.160 (0.844–1.594) | 0.359 | 1.231 (0.886–1.711) | 0.216 |  |
|  |  | Recessive^*^ | TT + GT | 342 (94.21) | 334 (96.81) | 1.0 (Ref.) | – | 1.0 (Ref.) | – | 957.172 |
|  |  |  | GG | 21 (5.79) | 11 (3.19) | 1.864 (0.885–3.927) | 0.101 | 2.074 (0.962–4.472) | 0.063 |  |
|  | rs9994887 | Codominant | AA | 92 (25.34) | 90 (26.09) | 1.0 (Ref.) | – | 1.0 (Ref.) | – | 962.284 |
|  |  |  | GA | 184 (50.69) | 177 (51.30) | 1.017 (0.712–1.452) | 0.926 | 1.055 (0.730–1.525) | 0.776 |  |
|  |  |  | GG | 87 (23.97) | 78 (22.61) | 1.091 (0.716–1.664) | 0.685 | 1.170 (0.756–1.811) | 0.480 |  |
|  |  | Additive^*^ | AA vs GA vs GG | |  | 1.044 (0.846–1.289) | 0.690 | 1.081 (0.869–1.345) | 0.483 | 960.311 |
|  |  | Dominant | AA | 92 (25.34) | 90 (26.09) | 1.0 (Ref.) | – | 1.0 (Ref.) | – | 960.572 |
|  |  |  | GA + GG | 271 (74.66) | 255 (73.91) | 1.040 (0.742–1.457) | 0.821 | 1.090 (0.769–1.545) | 0.630 |  |
|  |  | Recessive | AA + GA | 276 (76.03) | 267 (77.39) | 1.0 (Ref.) | – | 1.0 (Ref.) | – | 960.365 |
|  |  |  | GG | 87 (23.97) | 78 (22.61) | 1.079 (0.761–1.529) | 0.669 | 1.129 (0.788–1.619) | 0.508 |  |
|  | rs13112099 | Codominant | TT | 206 (56.75) | 194 (56.23) | 1.0 (Ref.) | – | 1.0 (Ref.) | – | 962.530 |
|  |  |  | GT | 82 (22.59) | 73 (21.16) | 1.058 (0.730–1.534) | 0.767 | 1.064 (0.725–1.560) | 0.751 |  |
|  |  |  | GG | 75 (20.66) | 78 (22.61) | 0.906 (0.624–1.315) | 0.602 | 0.941 (0.640–1.382) | 0.755 |  |
|  |  | Additive | TT vs GT vs GG | |  | 0.963 (0.803–1.155) | 0.686 | 0.980 (0.813–1.182) | 0.835 | 960.761 |
|  |  | Dominant | TT | 206 (56.75) | 194 (56.23) | 1.0 (Ref.) | – | 1.0 (Ref.) | – | 960.804 |
|  |  |  | GT + GG | 157 (43.25) | 151 (43.17) | 0.979 (0.727–1.318) | 0.890 | 1.001 (0.736–1.360) | 0.996 |  |
|  |  | Recessive^*^ | TT + GT | 288 (79.34) | 267 (77.39) | 1.0 (Ref.) | – | 1.0 (Ref.) | – | 960.630 |
|  |  |  | GG | 75 (20.66) | 78 (22.61) | 0.891 (0.623–1.275) | 0.529 | 0.925 (0.639–1.338) | 0.677 |  |
| Genotype distribution is presented as numbers (%) *AIC*: Akaike information criterion  ^*^ The best model for the genetic variant according to the Akaike information criterion (AIC).  ^a^ Adjusting for age, occupation, education level, marital status, age of smoking onset and pack-year. | | | | | | | | | | |
